# The InSiGHT approach to classification of mismatch repair gene variants

**DOI:** 10.1186/1897-4287-10-S2-A72

**Published:** 2012-04-12

**Authors:** JP Plazzer

**Affiliations:** 1 The Royal Melbourne Hospital, Australia

## 

The International Society for Gastrointestinal Hereditary Tumours (InSiGHT) is committed to the sharing of MMR variant information through the publicly accessible InSiGHT database. This amalgamation of various types of data related to MMR variants and Lynch Syndrome encompasses family history, tumour pathology, genotype, RNA, in silico and in vitro information sourced from published literature and submissions from various centers. InSiGHT is collaborating with organisations including those with national MMR data-sets, in order to centralise and make public the full extent of Lynch Syndrome associated variants. It is also InSiGHT’s goal to increase the number of submissions from individual labs, and encourages scientists from around the world to participate in this effort.

To understand the clinical impact of these variants, InSiGHT is bringing together an international group of experts to develop qualitative classification rules and apply them to variants of uncertain significance. The application of these classification rules to variants is a work in progress, with input from specialists in a diverse range of fields, and will enable thorough analysis of the available data. This process will culminate in regular teleconferences of a panel of experts who will discuss each variant and reach consensus on the clinical significance. Subsequent publication of the outcome will be of particular relevance to the medical genetics community.

